# Awareness, apathy, and depression in Alzheimer's disease and mild cognitive impairment

**DOI:** 10.1002/brb3.661

**Published:** 2017-03-06

**Authors:** Jean‐Pierre Jacus

**Affiliations:** ^1^ Consultations MémoireCentre Hospitalier du val d'AriègeFoix CedexFrance; ^2^ Laboratoire Epsylon, EA 4556Université Paul‐ValeryMontpellierFrance

**Keywords:** Alzheimer's disease, apathy, awareness, depression, mild cognitive impairment

## Abstract

**Introduction:**

Results from studies on awareness disorders in Alzheimer's disease (AD) are controversial because the methodologies, the “objects” of awareness, and the patients' pathologic stage all vary. Our study aimed to compare scores and correlates of awareness according to the stage of the disease and the assessment method.

**Methods:**

We compared 20 mild AD patients to 20 mild cognitive impairment (MCI) patients, using the *Patient Competency Rating Scale* (PCRS; patient vs. caregiver report) and the *Self‐Consciousness Scale* (rating scale). All patients underwent cognitive, psycho‐affective and behavioral assessments (global cognition, executive functions, episodic memory, anxiety‐depression, and apathy measures).

**Results:**

Groups were matched for age, education, and gender. They were comparable on the depression, anxiety, apathy and awareness scales (*p*s > .05), and differed for all cognitive variables (*p* < .05). Using the median split approach, greater apathy and lower depression were associated with poorer awareness on the *Self‐Consciousness Scale* (respectively: odds ratio [OR] = 4.8, *p *=* *.03; OR = 4.84, *p *=* *.04), and the PCRS (only apathy: OR = 9.3, *p *=* *.003). Greater apathy plus lower depression were associated with poorer awareness in both scales (PCRS: OR = 40.5, *p *=* *.005; Self‐consciousness scale: OR = 28, *p *=* *.012).

**Conclusion:**

These results evidence comparable awareness between AD and MCI patients. The correlates were more affective and behavioral than cognitive, independently from assessment method.

## 1 Introduction

One of the core features of Alzheimer's disease (AD) is the inability to perceive impairments and disturbances occurring in the course of the disease. There is great inter‐individual variability in the manifestations of this phenomenon. It seems to increase as the disease progresses and may affect the awareness of existence itself (Gil et al., 2001; Starkstein, 2014; Vogel, Boch Waldorff, & Waldemar, 2010). In the early stage of the disease, this inability can affect either distinct cognitive domains, or components relevant for daily living. It can prematurely affect the autonomy of patients because of their inability to avoid dangers for themselves or others, to accept help and care, leading them to an early placement in a structured living environment (Horning, Melrose, & Sulzer, 2014; Starkstein, 2014; Verhülsdonk et al., 2013).

Different terms referring to different conceptualizations have been used to describe this phenomenon (unawareness, anosognosia, denial, loss of self‐consciousness, lack of insight) and are often used interchangeably. According to Marková's (2005) conceptualization, we propose to use the term “awareness” in this paper. In fact, authors have suggested that awareness is the narrower form of insight (“the insight in dementia”) based only on the person's perception of impairments. In contrast, insight in a broader form (“the insight in psychiatric disorders”) considers the nature of the awareness from the judgments that patients express about their mental symptoms/disorders. This implies that awareness (narrower form) refers to a direct appraisal of impairment of functions (the “objects” of awareness), and is evaluated only quantitatively. In contrast, insight (wider form) refers to direct and indirect appraisals of mental changes (the “objects” of insight), evaluated quantitatively and qualitatively.

Awareness can be assessed in three possible approaches (Starkstein, 2014; Starkstein et al., 2006): (1) performance discrepancies; (2) discrepancy between caregiver and subject ratings; and (c) clinical evaluation. There are limitations for each method: (1) the ecological value of performance discrepancy measures, based on patient report on levels of performance on a given neuropsychologic task, remains unknown. (2) patient‐caregiver assessment discrepancies have shown that caregiver ratings may be influenced by burden, stress and depression. Nevertheless, this remains the most reliable and the best procedure (Starkstein, 2014). (3) Clinical ratings of patient awareness of illness are limited by the non‐structured nature of the evaluation, the lack of standardized diagnostic criteria and the unknown validity and reliability of this procedure. Although clinical ratings can be subjective and lack validity, they can take into account patient opinions on their impairments and illness. This enables more qualitative components of awareness to be captured providing a broader approach to this phenomenon in line with Marková's conceptualization.

The numerous studies on awareness in AD have yielded varied and contrasted results. The frequency of unawareness is reported to range between 20% and 80% by Starkstein (2014), and from 23% to 73% by Antoine et al. (2004). There are also as many studies reporting awareness disorders in AD, as studies not reporting these disturbances (Marková, 2005). There is however a better consensus to suggest that unawareness becomes more frequent as dementia progresses (Starkstein, 2014). Among the factors explaining these results, heterogeneity of assessment methods, different “objects” of awareness, and inclusion of patients at different stages of the disease are probably predominant (Marková, 2005; Starkstein, 2014). Nevertheless, contrasted results are also observed in mild cognitive impairment (MCI), thought to be a preclinical stage of AD (Petersen, 2004), or to be likely to progress to a full dementia syndrome (Roberts et al., 2014). Some studies have reported awareness impairment in MCI patients compared to healthy controls (HCs), or comparable unawareness with that of AD patient (Galeone et al., 2011; Tabert et al., 2002; Vogel et al., 2004), whereas other studies have reported preserved awareness in MCI (Kalbe et al., 2005; Zamboni et al., 2013). Alongside, a recent meta‐analysis reported that MCI patients have knowledge of their neuropsychologic deficits and that levels of awareness vary according to cognitive status, language, and memory abilities (Piras et al., 2016).

There are also varied results concerning the correlates of awareness in AD. The main correlates seem to be behavioral disturbances (Harwood, Sultzer, & Wheatley, 2000; Starkstein et al., 1995), in particular apathy (Horning et al., 2014; Starkstein et al., 2010; Vogel et al., 2010). Associations with executive performances have also been underlined (Gil et al., 2001; Kashiwa et al., 2005; Orfei et al., 2010). Several studies have highlighted associations with depression, greater depression being associated with better awareness (Conde‐Sala et al., 2013; Horning et al., 2014; Vogel et al., 2005), but these associations are not consistently reported (Vogel et al., 2010). Finally a recent study suggests that depression is associated with greater awareness, while behavioral disturbances (in particular apathy) are associated with impaired awareness (Horning et al., 2014).

In MCI, despite varied assessment methods, the correlates of awareness seem to be mainly cognitive (Kalbe et al., 2005; Orfei et al., 2008; Piras et al., 2016), suggesting that better awareness is associated with better neurocognitive abilities. These cognitive correlates in MCI are also congruent with Starkstein et al. (2006), reporting that awareness in patients with very mild dementia appeared significantly associated with anterograde memory and verbal comprehension deficits.

Our study aimed to compare the correlates of awareness in AD (cognitive vs. emotional/behavioral) and in MCI, according to the assessment method (clinical rating vs. patient‐caregiver report). Because the more pronounced is the dementia, the greater the unawareness is liable to be, we predicted that mild AD patients would exhibit more impaired awareness than MCI patients. Because different methods and different “objects” of awareness were implemented (see Section 2), we expected that the two awareness scales would not find the same results, nor the same correlates, depending on the stage of the disease.

## 2 Methods

### 2.1 Participants

Forty participants took part in the study: 20 patients with mild AD, and 20 patients with amnestic MCI (aMCI). All patients were recruited from a memory clinic (Centre Hospitalier du Val d'Ariège, France). The diagnosis of a MCI was made by a senior neurologist and a clinical neuropsychologist (J‐P J) using criteria defined by Petersen (2004), including: (1) memory complaint corroborated by an informant; (2) impaired episodic memory between −1.5 and −2 standard deviations from the population norm; (3) normal general cognitive function as determined by a normative *Mini Mental State Examination* (MMSE score; Folstein, Folstein, & Mc Hugh, 1975; Kalafat, Hugonot‐Diener, & Poitrenaud, 2003); (4) intact activities of daily living as determined by clinician judgment, and a structured interview with the patient and a caregiver; (5) and not meeting criteria for AD of the National Institute of Neurological and Communicative Disorders and Stroke/AD and Related Disorders Association—NINCDS‐ADRDA. The patients with AD were diagnosed according to the NINCDS‐ADRDA criteria and *the Clinical Dementia Rating Scale*. All patients underwent extensive medical and neurologic examinations, including nuclear magnetic resonance imaging, to ascertain the absence of any other major neurologic condition. All patients and caregivers gave informed consent for participation in the study.

### 2.2 Awareness measures

Awareness was assessed using two scales. (1) *The Patient Competency Rating Scal*e (PCRS) adopting the patient‐caregiver discrepancy approach, and (2)* the Self‐Consciousness Scale in AD* involving a clinical evaluation by structured interview with the patient.

*The Patient Competency Rating Scale* (Prigatano & Fordyce, 1986) requires patient and caregiver to complete the same 30 questions on the patient's ability to perform a variety of simple and complex tasks. These 30 questions are divided into four domains of competence: *Activities of daily‐living*,* Cognition*,* Interpersonal relations*, and *Emotion*. The patient and his/her caregiver are asked to judge how easy or how difficult a certain activity is. Items in the scale are scored from 1 to 5: 1 = *can't do*, 2 = *very difficult to do*, 3 = *can do, but with difficulty*, 4 = *fairly easy to do*, and 5 = *can do with ease*, with higher scores indicating greater competency. These domains all involve impairment of functions. The Awareness‐PCRS total score is obtained by subtracting the Patient‐PCRS score from the Caregiver‐PCRS score. Here, a PCRS total score below zero indicated poorer awareness of impairment of functions.

*The Self‐Consciousness Scale in AD* (Gil et al., 2001) is a structured interview with the patient including fourteen questions in seven domains: *identity* (questions nos. 1, 5–7), *knowledge of cognitive disturbances* (questions nos. 2–4), *affective state* (question no. 8), *representation of the body* (questions nos. 9, 10), *prospective memory* (question no. 11), *capacities for introspection* (question no. 12), and *moral judgments* (questions nos. 13, 14). Among these seven domains, *knowledge of cognitive disturbances* and *identity* mainly refer to impairment of function while the other domains concern judgments about mental, affective, or bodily states or moral values. This scale consequently involves different “objects” of awareness. A guideline is proposed by the authors for scoring patient responses. The greater the awareness the better is the score (maximum 28). Here, a total score below the median score for the whole sample indicated poorer awareness.

### 2.3 Cognitive, psycho‐affective and behavioral assessment

All patients underwent cognitive assessments using the MMSE (Folstein et al., 1975) measuring global cognition, the *Frontal Assessment Battery* (FAB; Dubois et al., 2000) assessing executive functions (two MCI and one AD patients did not perform the FAB), and the *free‐cued recall test* (Ergis, Van Der Linden, & Deweir, 1994) evaluating episodic memory (two AD patients did not perform this task). Immediate free recalls, immediate cued recalls, delayed free recall, and delayed cued recall were the four dependent variables for this task. For all the cognitive tasks, a higher score indicates better cognitive performances.

The psycho‐affective assessment was performed using the *Beck Depression Inventory* (BDI‐II; Beck et al., 1996) and the *State‐Trait Anxiety Inventory* (STAI; Spielberger, Gorsuch, & Lushene, 1970). These questionnaires are patient‐completed and higher scores indicates greater depression and/or anxiety.

The behavioral assessment used the *Apathy Evaluation Scale* (AES), completed by the patient, the caregiver, and the clinician (three versions; Marin, Biedrzycki, & Firinciogullari, 1991). A higher score indicates lesser apathy.

Before presenting the statistical analysis and our results, it is important to note that we formed a HC group. The control subjects were recruited from a pool of adult participants and senior‐citizen associations. They had no history of neurologic diseases or psychiatric disorders. They had a MMSE score above the 10th percentile taking into account their levels of education (Kalafat et al., 2003).

Nevertheless the use of this group in our study was not appropriate for three reasons:

- The *Self‐Consciousness Scale in AD* was inappropriate for a non‐clinical population. The only way to score this population correctly would be to attribute the best score (28/28) which is open to criticism, because the control subjects systematically obtain the top scores. This can induce associations between awareness and the different factors chosen to distinguish the control group from the others, for instance cognitive functions as measured by the MMSE.
- The PCRS was however better suited to the control group and there was no statistical difference between the three groups (HC, MCI and AD groups) for either the PCRS total score or the different subscale scores (all *p*s > .05 with the Kruskal–Wallis test).
- Finally, there was no measure of episodic memory in the HC group.

Therefore, we chose to compare the MCI and mild AD groups directly, knowing that they were comparable with control subjects for the PCRS, which was the most reliable measure of awareness for the control population.

### 2.4 Statistical analyses

The statistical analyses were carried out using the Statistical Package for Social Sciences (SPSS .20). Data were examined for normal distribution (tested using the Shapiro–Wilk test). As most of the demographic and clinical variables were not normally distributed (*p* < .05), we used non‐parametric statistics (Wilcoxon test [*W*] and Spearman's rank correlation coefficient [ρ]). Because most tasks did not have cutoff scores and because of the comparability of groups on these tasks, we used the median‐split approach. In this way, a score below the median score of the whole sample indicated a poorer performance, except for the PCRS score where the cut‐off was zero as indicated above. Odds ratios (OR) and their confidence intervals (CI 95%) were estimated using a conditional logistic regression model. The level of significance was set at *p *<* *.05, except for correlations where the level of significance was set at *p *<* *.01 because of their number (20 correlations).

## 3 Results

### 3.1 Demographic and clinical data

The AD and MCI patients were comparable for age, sex, and years of education (all *p*s > .05; Table 1).

**Table 1:** Demographic and clinical data

Variables median (min–max value)	MCI patients (*n* = 20)	AD patients (*n* = 20)	Statistics	*p*‐Values
Demographic				
Age	78.5 (61–83)	80.5 (71–90)	*W* = 352.5	.12
Gender, % females	55	50	χ^2^ = 0.1	.75
Years of education	8 (6–16)	8.5 (6–17)	*W* = 389.5	.57
Cognitive				
MMSE	25.5 (24–29)	24 (21–29)	***W*** ** = 323**	**.017**
FAB	15 (9–18)	13 (7–18)	***W*** ** = 295.5**	**.04**
Immediate free recall	20 (9–32)	10 (1–18)	***W*** ** = 204.5**	**<.0001**
Immediate cued recall	41 (34–48)	29.5 (17–38)	***W*** ** = 181**	**<.0001**
Delayed free recall	7.5 (1–16)	2 (0–8)	***W*** ** = 206.5**	**<.0001**
Delayed cued recall	14 (12–16)	9 (3–14)	***W*** ** = 189.5**	**<.0001**
Affective				
Depression (BDI‐II)	11 (0–26)	12.5 (0–27)	*W* = 401.5	.82
STAI (form state)	27 (20–51)	32.5 (20–48)	*W* = 370.5	.28
STAI (form trait)	42.5 (23–58)	42 (23–59)	*W* = 408	.96
Apathy				
AES (completed by patient)	57.5 (41–69)	55 (35–64)	*W* = 396	.70
AES (completed by caregiver)	45 (19–70)	49 (29–69)	*W* = 388	.55
AES (completed by clinician)	53.5 (41–66)	49 (38–61)	*W* = 370	.28
Awareness				
PCRS total score	−4 (−58 to 18)	−1.5 (−39 to 19)	*W* = 390	.59
Self‐consciousness total score	23.5 (18–27)	23 (18–27)	*W* = 374.5	.33

AD patients, Alzheimer's disease patients; AES, Apathy Evaluation Scale; FAB, Frontal Assessment Battery; MCI patients, Mild Cognitive Impairment patients; MMSE, Mini Mental State Examination; PCRS, Patient Competency Rating Scale; STAI, State Trait Anxiety Inventory.

The groups differed for MMSE (global cognition), FAB (executive functions), and *free‐cued recall test* (episodic memory) scores. The AD patients had lower MMSE scores (*W* = 323, *p *=* *.017), lower FAB scores (*W* = 295.5, *p *=* *.04), lower immediate free recall scores (*W* = 204.5, *p *<* *.0001), lower immediate cued recall scores (*W* = 181, *p *<* *.0001), lower delayed free recall scores (*W* = 206.5, *p *<* *.0001), and lower delayed cued recall scores (*W* = 189.5, *p *<* *.0001), than the MCI patients.

The groups were comparable for psycho‐affective scores (BDI‐II and STAI), and apathy (AES completed by the patient, the caregiver and the clinician; all *p*s > .05). They were also comparable for awareness as measured by the PCRS total score, and the *Self‐Consciousness Scale* total score (all *p*s > .05).

### 3.2 Correlates of awareness (in the whole sample)

The PCRS total score showed significant correlations with the AES completed by the caregiver (ρ = .576, *p *<* *.0001), the BDI‐II score (ρ = .436, *p *=* *.005), and the STAI‐trait score (ρ = .408, *p *=* *.009). The PCRS score showed no significant association with cognitive performances in either the whole sample or the different groups (because of cognitive differences between groups; all *p*s > .01).

The *Self‐Consciousness Scale* total score also showed significant correlations with the AES completed by the caregiver (ρ = .461, *p *=* *.003), and a statistically non‐significant trend for the STAI‐trait score (*p* = .016). The Self‐Consciousness score showed no significant association with cognitive performances for either the whole sample or the different groups (all *p*s > .01).

A comprehensive analysis of *Self‐consciousness scale* correlates showed that only *knowledge of cognitive disturbances* correlated with the STAI‐trait (ρ = .459, *p *=* *.003) and the AES completed by the caregiver (ρ = .417, *p *=* *.007), while the four domains of the PCRS correlated with apathy and/or psycho‐affective scores.

No correlation was noted between the AES scores (three versions) and the BDI‐II or the STAI state or trait scores (all *p*s > .01). Finally, a statistically non‐significant association was observed between the two awareness scales total scores (*p *=* *.013). A comprehensive analysis of correlations between the two scales showed that *knowledge of cognitive disturbances* in Self‐consciousness correlated with the PCRS total score (ρ = .465, *p *=* *.003), PCRS *Activities of daily‐living* (ρ = .490, *p *=* *.001) and PCRS *Cognition* (ρ = .496, *p *=* *.001). This analysis also showed that *representations of the body* in Self‐consciousness correlated with PCRS *Interpersonal relations* (ρ = −.411, *p *=* *.008).

### 3.3 Associations between awareness, apathy, and depression

#### 3.3.1 Awareness and apathy

The percentage of patients presenting greater apathy assessed by the caregiver (defined by an AES score below the median score of 48/72) was significantly larger among patients with a poorer total awareness score on the PCRS (defined by a negative score; 72.7% vs. 27.3%, OR = 9.3, 95% CI = 2.18–39.96, *p *=* *.003; Table 2). Similarly, the percentage of patients presenting greater apathy assessed by the caregiver was significantly larger among patients with a poorer total awareness score on the *Self‐Consciousness Scale* (defined by a score below the median score of 23/28; 70.6% vs. 29.4%, OR = 4.8, 95% CI = 1.14–20.8, *p *=* *.03).

**Table 2:** Percentage of patients with greater apathy scored by the caregiver, lower depression and greater apathy plus lower depression according to the awareness profile: odds ratio, *p*‐values and confidence intervals

	Awareness profile		OR	*p*‐Value	95% CI
	PCRS < 0 Poorer awareness (%)	PCRS > 0 Better awareness (%)			
Greater apathy assessed by the caregiver (<48/78)	72.7	22.2	9.3	.003	2.18–39.96
Lower depression (<11/63)	60	40	—	.09	—
Greater apathy plus lower depression (*n* = 21)	81.8	10	40.5	.005	3.09–530.29

	Self‐conscious. <23/28 Poorer awareness (%)	Self‐conscious. >23/28 Better awareness (%)			
Greater apathy assessed by the caregiver (<48/78)	70.6	33.3	4.8	.03	1.14–20.8
Lower depression (<11/63)	68.8	31.2	4.84	.04	1.08–21.58
Greater apathy plus lower depression (*n* = 21)	87.5	20	28	.012	2.07–379.25

PCRS, Patient Competency Rating Scale; Self‐conscious, Self‐Consciousness Scale.

#### 3.3.2 Awareness and depression

The percentage of patients scoring low for depression (defined by a BDI‐II score below the median score of 11/63) was significantly larger among patients with a poorer total score on the *Self‐Consciousness Scale* (68.8% vs. 31.2%, OR = 4.84, 95% CI = 1.08–21.58, *p *=* *.04). The association between the BDI‐II score and the PCRS total score failed to reach significance (60% vs. 40%, *p *=* *.09).

There was no association between STAI‐trait (cut‐off was the median score for the whole sample [42/80]) and the PCRS total score or the Self‐Consciousness total score (all *p*s > .05).

#### 3.3.3 Awareness, apathy, and depression

Finally, the percentage of patients scoring lower for depression and higher for apathy on caregiver assessment was significantly larger among patients with poorer total awareness scores on the PCRS (81.8% vs. 18.2%, OR = 40.5, 95% CI = 3.09–530.29, *p *=* *.005), or with poorer total awareness scores on the *Self‐Consciousness Scale* (87.5% vs. 12.5%, OR = 28, 95% CI = 2.07–379.25, *p *=* *.012; comparison with patients scoring higher for depression and lower for apathy on caregiver assessment).

## 4 Discussion

The main results of this study show (1) that the groups were comparable on the two awareness scales and (2) that the correlates of awareness were mainly affective and behavioral irrespective of the stage of the disease (MCI vs. mild AD) and irrespective of the assessment method (clinical rating vs. patient‐caregiver report).

### 4.1 Comparability of the groups on the two awareness scales

The groups were comparable on the two awareness scales. These results are not congruent with our first hypothesis predicting that mild AD patients would be more impaired because awareness decreases as dementia progresses. Nevertheless, results concerning the comparison between MCI and AD on awareness are known to be controversial (see Section 1), and Galeone et al. (2011) like Vogel et al. (2004) have already reported the comparability between MCI and AD patients. A recent study carried out by Rios‐Silva et al. (2016) also reported non‐significant differences between two measures of awareness in a follow‐up study over a mean period of 24 months, in particular for MCI patients converting to mild AD at follow‐up . Our results are congruent with these studies and can be explained by the correlates of awareness reported using the two scales.

### 4.2 Correlates of awareness in our study

Alzheimer's disease patients were more impaired than MCI patients for all cognitive variables. This can be explained by the Petersen (2004) criteria used to recruit MCI patients. AD and MCI patients were however comparable on all psycho‐affective and behavioral (apathy) variables. Awareness scores showed no correlations with cognitive functions, contrasting with the main results reported in MCI (Kalbe et al., 2005; Orfei et al., 2008; Piras et al., 2016), as in very mild AD (Starkstein et al., 2006). However, our results show strong correlations between awareness and apathy, and between awareness and anxiety‐depression (in the whole sample, because of the comparability of the groups for these variables). These results are congruent with those of Starkstein et al. (2010) and Vogel et al. (2010) suggesting that behavioral disturbances, and in particular apathy, are the main correlates of awareness, or can be predicted by awareness in AD. Our results are also especially congruent with those of Horning et al. (2014), reporting that awareness significantly predicts depressed mood, anxiety, and apathy in AD. The comparability between groups regarding psycho‐affective and behavioral variables and the associations between these variables and awareness could explain the comparability between groups on the two awareness scales.

Moreover, despite differing methodologies, these correlates were evidenced with both awareness scales: apathy and anxiety‐depression with the PCRS total score, apathy and anxiety (statistically non‐significant trend) with the total *Self‐consciousness scale* score. Similarly, the awareness scales showed a tendency towards correlations with each other (total scores). Our second hypothesis, that scores and correlates between awareness scales would differ because of differing assessment methods, seems not to hold. Nevertheless, as illustrated by the comprehensive analysis of Self‐consciousness correlates, the two scales identified the same correlates with the same “objects” of awareness (impairment of functions). These results are congruent with Marková' (2005) analysis of the main role played by the “objects of insight” to explain the heterogeneity of results for awareness in dementia. In our study, the assessment methodology did not appear very relevant for the different correlates, but the “objects” of awareness did, because only “objects” concerning impairment of functions had the same correlates, and not other “objects” such perception of affective state or moral judgments. Our second hypothesis is therefore not totally invalidated.

The core feature of our results is the importance of affective symptoms and apathy as correlates of awareness evaluated with two different methodologies. Firstly, we observed strong associations between poorer awareness and greater apathy (with both awareness scales), between poorer awareness and lower depression (with the PCRS) and finally between poorer awareness and greater apathy plus lower depression (with both awareness scales). Secondly, we observed that apathy and anxiety or depression were not correlated with one another. These results are fully congruent with those of Horning et al. (2014), suggesting that awareness may be differentially related to affective symptoms and apathy within AD, such that patients with better awareness are more depressed or anxious, whereas patients with poorer awareness are more apathetic.

Interestingly, we observed that only apathy completed by the caregiver showed strong correlations with the two awareness scales. In fact the majority of patients in both clinical groups (1/4) underestimated their apathy compared to the caregiver rating (the mean difference between the self‐rating and the caregiver rating was −8/72). This could be explained by unawareness of apathy in MCI as in mild AD. Unawareness in AD is not only related to cognitive symptoms but also to affective symptoms as highlighted by Verhülsdonk et al. (2013). Thus unawareness could also be related to apathetic symptoms as reported by Robert et al. (2002). In both clinical groups, the underestimation of apathy between the self‐rating and the clinician rating was lower (the mean difference was −2/72). This could be explained by the fact that the clinician rating is based on an interview with the patient with whom the clinician is less well acquainted than the caregiver. These results suggest the need to include more items concerning apathetic and affective symptoms in awareness scales for Alzheimer's patients.

From a neuroanatomic point of view, various studies have reported a significant association between awareness and hypo‐perfusion/hypo‐metabolism in several brain regions such the frontal cortex (including the orbitofrontal, frontal dorsal and cingulate cortices), the inferior parietal cortex, and the medial temporal regions (Starkstein, 2014). Neuroimaging research has also consistently underlined the involvement of the anterior cingulate in patients with apathy (Marshall et al., 2006, 2007). Depression has been found to be significantly linked to the orbitofrontal cortex in elderly patients, whereas associations with the anterior cingulate and temporal cortices are less consistently reported (Benoit & Robert, 2007).

Awareness thus involves various substrates, including the frontal, inferior parietal and medial temporal cortices. Apathy and depression seem to have common frontal substrates with awareness, and distinct substrates between themselves (respectively the cingulate and orbitofrontal cortices). Our results evidencing associations between awareness and apathy, awareness and depression but not between apathy and depression, are coherent with these neural bases.

From a clinical viewpoint, while several studies have found increasingly impaired awareness in AD to be associated with less severe depression, Migliorelli et al. (1995) reported that only minor depression was associated with better awareness, suggesting an emotional reaction to the awareness of deficits (Starkstein, 2014). Our results using the median split approach (applied to depression and other variables) are also congruent with this finding.

There is convincing evidence to support the notion that later‐life major depression can be seen as a prodrome of dementia (Bennett & Thomas, 2014), whereas apathy is thought to be the best behavioral predictor of transition from MCI to dementia (Geda et al., 2007, 2008; Sobow et al., 2010). One question is whether awareness disorders lie on a continuum from depression to apathy in both AD and MCI (i.e., from perceiving difficulties and showing concern to not perceiving these difficulties and therefore showing no concern). This hypothesis however needs further research comparing HCs and patients with depression, apathy, and dementia, using a longitudinal methodology and neuroimaging techniques. Up to now, few studies have used a follow‐up methodology to clarify the development of unawareness in AD as the disease progresses (Rios‐Silva et al., 2016; Starkstein et al., 2010).

## 5 Limitations and Conclusion

Our study is not exempt from limitations. First, the small sample means that our results remain exploratory. Also, the AD and MCI groups were comparable on affective symptoms reported by the patients, whereas apathy was also assessed by the caregivers and the clinician. It is known that unawareness can be related not only to deficits in cognition, but also to affective symptoms such as depression and anxiety (Verhülsdonk et al., 2013). The comparability of our groups on the psycho‐affective scales is thus open to discussion. The difficulty of including a control group using a clinical rating and the absence of a measure of episodic memory in this group are also major limitations, restricting the study to a comparison between two clinical groups. Nevertheless, the three groups were comparable on awareness measured by the PCRS. Finally, our results indicate strong associations between awareness, apathy, and depression, but no direction. Possibly poorer awareness significantly increases the risk of greater apathy and lower depression (OR = 40.5 for PCRS and 28 for *Self‐consciousness scale*), or possibly the reverse is true. The first option appears more congruent with Starkstein et al.'s (2010) longitudinal study, reporting that patients with awareness impairments had a significantly greater increment on apathy scores over the time compared to patients with preserved awareness. This is also in line with Horning et al. (2014), who showed that awareness levels predict affective and apathy scores. However no causality between awareness and apathy, or between awareness and affective symptoms, can be deduced from our results.

In summary, these results underline the comparability of awareness between AD and MCI patients, and show that the correlates are more affective and behavioral than cognitive. Awareness and its correlates are also comparable across assessment methods, but seem to be related to the “objects” of awareness. Additional research on awareness in AD and MCI should continue to explore its correlates, and look for causal relationships. Similarly, the design of awareness scales should include items covering psycho‐affective and apathetic symptom profiles.

## Conflict of Interest

The authors declare that there is no conflict of interests regarding the publication of this paper.
